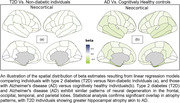# Type 2 diabetes and Alzheimer's disease share common cortical neurodegenerative patterns

**DOI:** 10.1002/alz.087602

**Published:** 2025-01-09

**Authors:** Mahboubeh Motaghi, Olivier Potvin, Simon Duchesne

**Affiliations:** ^1^ Laval University, Quebec City, QC Canada; ^2^ Centre de recherche de l’Institut Universitaire de Cardiologie et de Pneumologie de Québec, Quebec city, QC Canada; ^3^ Centre de recherche de l’Institut Universitaire de Cardiologie et de Pneumologie de Québec, Quebec, QC Canada; ^4^ Université Laval, Quebec City, QC Canada

## Abstract

**Background:**

Type 2 diabetes (T2D) is a prevalent health condition associated with cognitive impairment and dementia. T2D induces adverse effects not only on the pancreas but also on the liver, kidneys, muscles, fat cells, and, notably, the brain. Both T2D and Alzheimer's disease (AD) exhibit associations with neurodegeneration, yet the extent of their shared patterns of brain atrophy remains poorly understood, potentially indicating common pathways. The primary objectives were to assess the similarity between neural degeneration patterns in T2D and AD and to identify commonalities between these two diseases.

**Method:**

We employed linear models to compare neurodegeneration patterns linked to T2D and AD. A total of 43,172 participants from the United Kingdom Biobank (UKBB) (3,479 with T2D) and 8,378 from ADNI (871 with AD) were included in our study. We selected Free Surfer‐derived cortical thickness measures extracted from T1‐weighted MRI, age, sex, and blood biomarkers of T2D. The age range for UKBB was 40‐70 years, while for ADNI, it was 55‐97 years. We filtered data for individuals aged 55 and older, normalized features, and created age‐ and sex‐matched groups. We then created cortical atrophy maps using linear regression models for each group. Comparison of the AD and T2D maps was performed using permutation‐based tests accounting for spatial autocorrelation.

**Result:**

Our findings revealed similar patterns of neural degeneration in T2D and AD brain maps, particularly in the frontal, parietal, temporal, and occipital lobes, suggesting shared neuroanatomical alterations. Statistically significant overlap was observed in atrophy patterns between AD and T2D patients. T2D individuals exhibited greater atrophy in brain regions affected in AD, such as the hippocampus. Permutation analysis indicated no significant difference between the impact of T2D and AD on brain regions, supporting the notion that T2D is a substantial risk factor for AD.

**Conclusion:**

This study enhances our understanding of the similarities in how T2D and AD impact the brain, highlighting the potential connection between these conditions. Further research is warranted to explore underlying mechanisms and implications for clinical interventions.